# Assessment of the relationship between tooth inclination and gingival and alveolar bone dimensions using computed tomography of the maxillary anterior teeth: a cross-sectional study

**DOI:** 10.1590/2177-6709.27.4.e222136.oar

**Published:** 2022-09-23

**Authors:** Katia MONTANHA-ANDRADE, Ieda Margarida CRUSOÉ-REBELLO, Mauricio BARRETO, Frederico Sampaio NEVES, Jean Nunes dos SANTOS, Patricia Ramos CURY

**Affiliations:** 1 Universidade Federal da Bahia, Faculdade de Odontologia (Salvador/BA, Brazil).; 2 Universidade Federal da Bahia, Faculdade de Odontologia, Departamento de Radiologia Dentomaxilofacial (Salvador/BA, Brazil).; 3 Escola Bahiana de Medicina e Saúde Pública, Departamento de Implantologia (Salvador/BA, Brazil).; 4 Universidade Federal da Bahia, Faculdade de Odontologia, Departamento de Patologia Oral (Salvador/BA, Brazil).; 5 Universidade Federal da Bahia, Faculdade de Odontologia, Departamento de Periodontia (Salvador/BA, Brazil).

**Keywords:** Alveolar process, Gingiva, Periodontics, Orthodontics, Tomography

## Abstract

**Objective:**

The present study aimed to investigate the relationship between tooth inclination and gingival and bone dimensions in maxillary anterior teeth.

**Methods:**

This cross-sectional study included cone-beam computed tomography (CBCT) images of 160 maxillary anterior teeth (30 individuals). Tooth inclination, gingival and bone thickness, and distances from cementoenamel junction to alveolar bone crest and gingival margin were measured in the labial surface. The correlations were analyzed using Pearson and partial correlation tests (*p*≤0.05).

**Results:**

In the central incisors, tooth inclination was positively and significantly related to apical bone thickness (R = 0.34, *p*= 0.001). In the canines, tooth inclination was negatively and significantly related to cervical bone thickness (R = - 0.34, *p*= 0.01) and positively associated to apical bone thickness (R = 0.36, *p*= 0.01) and to gingival margin-cementoenamel junction distance (R = 0.31, *p*= 0.03). In the lateral incisors, tooth inclination was not associated with gingival or bone dimensions.

**Conclusions:**

In the central incisors, the greater the labial tooth inclination, the greater is the apical bone thickness. In the canines, the greater the labial tooth inclination, the smallest is the cervical bone thickness, the greater is the apical bone thickness, and the greater is the gingival margin. Gingival and bone dimensions should be assessed when planning orthodontic treatment involving buccal movement of central incisors and canines.

## RESUMO

**Objetivo:**

O presente estudo teve como objetivo investigar a relação entre a inclinação dentária e as dimensões ósseas e gengivais em dentes anteriores superiores.

**Métodos:**

Esse estudo transversal incluiu imagens de tomografia computadorizada de feixe cônico (TCFC) de 160 dentes anteriores superiores divididos em três grupos (incisivo central, incisivo lateral e canino). Todos os pacientes tinham 18 anos ou mais, não tinham tratamento ortodôntico prévio nem história clínica que pudesse afetar as dimensões ósseas ou gengivais. A inclinação dentária, a espessura do osso e da gengiva e as distâncias da junção cemento-esmalte à crista óssea alveolar e à margem gengival foram medidas na face vestibular. As correlações foram analisadas por meio dos testes de Pearson e de correlação parcial (*p*≤0,05).

**Resultados:**

Nos incisivos centrais, a inclinação dentária foi positiva e significativamente relacionada à espessura do osso apical (R = 0,34, *p*= 0,001). Nos caninos, a inclinação dentária foi negativa e significativamente relacionada à espessura do osso cervical (R = - 0,34, *p*= 0,01) e positivamente associada à espessura do osso apical (R = 0,36, *p*= 0,01) e à distância entre a margem gengival e a junção cemento-esmalte (R = 0,31, *p*= 0,03). Nos incisivos laterais, a inclinação do dente não foi associada às dimensões do osso ou da gengiva.

**Conclusões:**

Nos incisivos centrais, quanto maior a inclinação vestibular do dente, maior a espessura do osso apical. Nos caninos, quanto maior a inclinação vestibular do dente, menor a espessura do osso cervical, maior a espessura do osso apical e maior a margem gengival. As dimensões ósseas e gengivais devem ser avaliadas ao se planejar o tratamento ortodôntico envolvendo a movimentação vestibular dos incisivos centrais e caninos.

## INTRODUCTION

Understanding the factors that determine gingival and alveolar bone thickness is crucial for successful orthodontic, periodontal, and prosthodontic therapies. Many factors have been suggested to affect gingival and alveolar bone thickness in individuals with a healthy periodontium, such as age and sex; facial growth pattern; tooth shape; and events that occur during tooth eruption, including tooth inclination.^1-8^ However, the impact of tooth inclination on gingival and alveolar bone thickness remains poorly understood. While some studies have reported that inclination or protrusion of the incisors is not related to gingival and bone thickness, others have observed an association between these factors.^9^ ^-^ ^16^ These controversial results may be attributed to differences and limitations in the methods used to measure tooth inclination, as well as gingival and alveolar bone dimensions. 

Tooth inclination has been evaluated using lateral cephalometry or cone-beam computed tomography (CBCT).^9^ ^-^ ^16^ However, lateral cephalograms only allow evaluation of the maxillary and mandibular central incisors. Overlap between images of the right and left sides is another limitation. On the other hand, when measurements are made on the sagittal slice of CBCT images, there is no overlap of structures, and the buccolingual inclination of each tooth can be measured. Three previous studies evaluated the association between periodontal tissue dimensions and tooth inclination measured on the sagittal slice of CBCT images. From these, two studies showed that retroclined maxillary central incisors have thinner supporting bone, while the other study found that proclined maxillary central incisors have greater apical bone thickness.^13^ ^,^ ^14^ ^,^ ^16^ Another study investigated the association between the inclination of all teeth and alveolar bone thickness (BT) in adults with Class III dentofacial deformities, compared to Class I^7^. This study showed that Class III group exhibited greater buccolingual inclination and thinner alveolar bone at the cervical and apical levels than the group with normal occlusion, although the correlation was weak.^7^ Another study did not measure BT, but assessed dehiscence and fenestration, and found that tooth inclinations were not related with the frequency of dehiscence.^17^

Gingival thickness (GT) has been assessed by means of many methods, including the transparency of the periodontal probe, ultrasonic devices, transgingival probes, and CBCT. Periodontal probe transparency through the free gingiva discriminates between thin and thick biotypes;^18^ however, this method does not quantify gingival thickness. Ultrasonic devices are not reliable when GT is >0.5 mm,^18^ ^-^ ^20^ while transgingival probes necessitate invasive measurements.^20^ In contrast, CBCT can be used to reliably quantify GT in a non-invasive manner.^21^ ^,^ ^22^ With regard to the relationship between the gingival biotype and tooth inclination, previous studies found no association between maxillary incisor proclination and the thin gingival biotype.^6^ ^,^ ^10^ ^,^ ^15^ However, none of those studies used CBCT images to measure gingival thickness. With regard to the level of the gingival margin (GM), although a few studies described a positive association between the presence or absence of gingival recession (clinically evaluated) and tooth inclination,^10^ ^,^ ^15^ to the best of our knowledge, the relationship between the GM level and tooth inclination has not been previously evaluated. 

Therefore, there is a lack of consensus among previous studies regarding the associations between tooth inclination and gingival and bone dimensions.^10^ ^,^ ^11^ ^,^ ^13^ ^-^ ^16^ Accordingly, the aim of this study was to investigate the relationship between tooth inclination and gingival and bone dimensions in the maxillary anterior teeth using CBCT, which allows highly accurate measurement of tooth inclination, as well as gingival and bone dimensions on the same image.^23^ ^,^ ^24^ The tested hypothesis was that there is a negative correlation between the inclination of the maxillary anterior teeth and the thickness of the buccal bone and gingiva.

## MATERIAL AND METHODS

The study protocol was independently reviewed and approved (protocol number: 1.759.719) by the Ethics Committee of *Escola Bahiana de Medicina e Saúde Pública* (Bahia, Brazil), and the study was conducted in accordance with the Helsinki declaration. An informed consent was obtained from all participants.

### STUDY DESIGN, SETTING, AND PARTICIPANTS

This cross-sectional study included a convenience sample of 30 individuals (160 teeth). The primary outcomes were gingival and bone thickness and height, with sex and age as potential confounders. 

The images were obtained from a CBCT image bank. For image inclusion, a list of patients was generated from an existing implant clinic database of patients who had undergone CBCT for diagnostic procedures between January 2017 and March 2019. The inclusion criteria were as follows: availability of maxillary anterior tooth images, and age > 18 years. The exclusion criteria were: previous orthodontic treatment; teeth with morphological anomalies; alveolar bone loss characterizing periodontal disease; previous surgical intervention in the anterior region of the maxilla; pregnancy; use of medications known to induce gingival growth; systemic diseases, including acquired immunodeficiency syndrome, diabetes mellitus, congenital disorders, and Crohn’s disease; and the presence of endodontic pathologies in the regions of interest or dental restorations extending beyond the cementoenamel junction (CEJ).

A total of 42 patients were screened for the inclusion and exclusion criteria. From these, 12 patients with a history of orthodontic treatment were excluded, and the remaining 30 were considered eligible for analyses. Eighteen teeth were absent. Eventually, 160 teeth, including 52 central incisors, 55 lateral incisors, and 53 canines, were analyzed. 

### CBCT SCANNING

At the time of CBCT scanning, the patients wore a plastic lip retractor, and images were acquired using a CS 8100 3D tomography device (Carestream Health Inc., Marne La Vallée, France). The acquisition protocol was in accordance with the manufacturer’s instructions and anatomical profile of the patient, with a voxel size of 0.15 mm and a field of view of at least 100 × 50 mm.^25^ ^,^ ^26^

### CBCT IMAGE ANALYSIS

A single examiner, specialized in Orthodontics, trained by an experienced radiologist, performed all measurements using CS 3D Imaging software (version 3.5.18; Carestream Health Inc., Marne La Vallée, France). After the training period, the intraexaminer correlation coefficient was at least 0.8 for all planned linear measurements.

Changes in brightness and contrast and the zoom function were used for better visual assessment.

**Figure 1:**
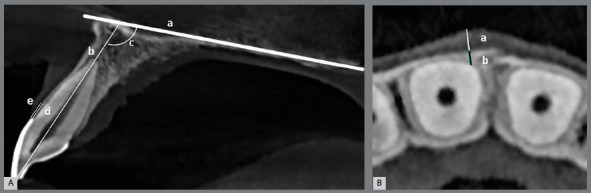
Cone-beam computed tomography images. **A**) Parasagittal image: (a) palatal line, between the anterior and posterior nasal spines; (b) long axis of the maxillary right central incisor; (c) tooth inclination in relation to the palatal line; (d) cementoenamel junction-alveolar crest distance; (e) gingival margin-alveolar crest distance. **B**) Axial image: bone (a) and gingival (b) thickness.

For the evaluation of buccolingual inclination, a palatal line was drawn from the anterior nasal spine to the posterior nasal spine on the sagittal images (Fig 1A). Subsequently, the angle between the long axis of each maxillary anterior tooth and the palatal line was measured (Fig 1A) on the best image generated in the sagittal view, without any change in the dental inclination on parasagittal or coronal sections.

Five dimensions were measured for all the anterior maxillary teeth (Figs 1A, B): (1) gingival margin height, measured as the distance from GM to cementoenamel junction (CEJ) at the center of the buccal surface (GM-CEJ); (2) bone height, measured as the distance from CEJ to the alveolar bone crest (BC) at the center of the buccal surface (CEJ-BC); (3) dentogingival unit, measured as the distance from GM to BC (GM-BC); (4) bone thickness (BT), measured as the thickness of the thinner buccal bone overlying the cervical, middle, and apical thirds of the roots; (5) and gingival thickness (GT), measured as the thickness of the thinner buccal gingiva overlying the cervical and middle thirds of the roots.

Briefly, the tooth was centered on the axial, coronal, and sagittal CBCT image slices. The vertical angulation was set according to the long axis at the center of the tooth of interest, and a slice location was positioned to pass through the vertical angulation, perpendicular to the alveolar ridge. Once the vertical and horizontal dimensions for analysis were determined, a sagittal section was created, and a horizontal reference line was drawn at the level of CEJ. The distance between CEJ and the tooth apex was measured, and the tooth root was divided into three segments. Then, the horizontal reference line placed at CEJ was used to determine the CEJ-BC and the GM-BC. These parameters were measured in the centered sagittal view of each tooth. The GM-CEJ was computed by subtracting CEJ-BC from GM-BC. BT and GT were measured in the axial view (Fig 1B). In the apical part, only bone was measured, because there was no attached mucosa. 

### STATISTICAL ANALYSIS

In total, 160 maxillary anterior teeth were analyzed. Each tooth was considered one experimental unit. Forty-two dimensions could not be obtained because of unclear sagittal images, and were omitted from the analysis. 

A descriptive analysis was performed to obtain average values for tooth inclination, BT, GT, CEJ-BC, GM-CEJ, and GM-BC, according to the tooth category (Central incisor, Lateral incisor, Canine). After testing for homogeneity of variances (Levene test), analysis of variance followed by the Tukey test was used for comparisons of measurements among the central incisors, lateral incisors, and canines, while *t*-tests were used for comparisons of gingival and bone measurements between male and female participants and between individuals aged <50 years and those aged ≥50 years.^26^

Pearson’s correlation analysis was used to evaluate the association between tooth inclination and the periodontal dimensions for each tooth group. When the differences between male and female participants or younger and older individuals were statistically significant (*p*≤ 0.05), a partial correlation controlling for sex and/or age was used. 

A significance level of 5% (α=0.05) was adopted for all statistical tests, which were performed using Statistical Package for the Social Sciences software, version 13.0 (SPSS Inc., Chicago, IL, USA). 

## RESULTS

### GENERAL CHARACTERISTICS

The study sample comprised 160 maxillary anterior teeth from 30 adult patients, including 19 women and 11 men, aged 18 to 66 years (mean ± standard deviation = 39.40 ± 12.06 years). 

The mean inclinations of the maxillary central incisors, lateral incisors, and canines were 112.10 ± 6.9° (range: 94° to 129°), 114.51 ± 6.4° (range: 99º to 126°), and 106.6 ± 6.2° (range: 87° to 119°), respectively, with statistically significant differences among the tooth groups (*p*< 0.001) (Table 1).

**Table 1:** Tooth inclination (degrees), and gingival and bone dimensions (mm) according to teeth (mean ± standard deviation).

	Central incisors / Tooth third			Lateral incisors / Tooth third			Canines / Tooth third		
	Cervical	Middle	Apical	Cervical	Middle	Apical	Cervical	Middle	Apical
Inclination	112.10±6.90*			114.52±6.32*			106.57±6.10		
BT	0.48±0.20*	0.53±0.34	0.31±0.31	0.46±0.26*	0.56±0.43	0.32±0.56	0.35±0.20	0.44±0.36	0.26 ± 0.28
GT	0.62±0.22*	0.61±0.20		0.51±0.27*	0.61±0.27		0.39±0.19	0.39±0.25	
CEJ-BC	1.93±0.68			2.10±1.40			2.33±1.06		
GM-CEJ	1.05±0.82			1.34±1.24*			0.74±1.11		
GM-BC	2.99±0.88			3.44±1.26			3.07±0.78		

* p ≤ 0.05 (compared to canines; Tukey test). BT = bone thickness, GT = gingival thickness, CEJ-BC = distance from cementoenamel junction to bone crest; GM-CEJ = distance from gingival margin to cementoenamel junction; GM-BC = distance from gingival margin to bone crest.

The mean BT in the cervical third were 0.48 ± 0.20, 0.46 ± 0.26, and 0.35 ± 0.20 mm for the central incisors, lateral incisors, and canines, respectively, with statistically significant differences among the tooth groups (*p*= 0.01). These values were not statistically different among the tooth groups for the middle third (*p*= 0.45) and the apical third (*p*= 0.77) (Table 1).

The mean GT in the cervical region were 0.62 ± 0.22, 0.51 ± 0.27, and 0.39 ± 0.19 mm for the central incisors, lateral incisors, and canines, respectively, with statistically significant differences among tooth groups (*p*< 0.001). In the middle third, these values were not statistically different from each other (*p*> 0.05, Table 1).

The mean CEJ-BC and GM-BC distances showed no significant differences among the tooth groups (*p*≥ 0.06), whereas the mean GM-CEJ was significantly greater for the lateral incisors (1.34 ± 1.22 mm) than for the canines (0.70 ± 1.11 mm, *p*= 0.01) (Table 1).

Men and women showed differences in the gingival and bone dimensions for the central incisors and canines. In the central incisor group, the bone in the cervical region was significantly thicker for women than for men (*p*= 0.01); moreover, the GM-CEJ was greater in women than in men (*p*= 0.04). In the canine group, the bone in the middle and apical regions was significantly thicker for men than for women (*p*≤ 0.02). The other differences were not statistically significant (Table 2).

**Table 2:** Gingival and bone dimensions (in mm) according to sex (mean ± standard deviation).

Dimensions	Central incisor		Lateral incisor		Canine	
	Female	Male	Female	Male	Female	Male
GM-BC	3.1 ± 0.75	2.8 ± 1.06*	3.41 ± 1.06	3.47 ± 1.51	2.30 ± 0.79	3.20 ± 0.76
CEJ-BC	1.84 ± 0.68	2.10 ± 0.67	1.90 ± 0.99	2.42 ± 1.87	2.22 ± 1.21	2.56 ± 0.78
GM-CEJ	1.24 ± 0.85	0.74 ± 0.68*	1.51 ± 1.32	1.05 ± 1.07	0.81 ± 1.05	0.64 ± 1.20
BT						
Cervical	0.54 ± 0.19	0.39 ± 0.17*	0.44 ± 0.24	0.48 ± 0.28	0.31 ± 0.22	0.40 ± 0.16
Middle	0.47 ± 0.28	0.63 ± 0.41	0.46 ± 0.31	0.70 ± 0.57	0.34 ± 0.36	0.63 ± 0.25*
Apical	0.28 ± 0.33	0.36 ± 0.28	0.26 ± 0.55	0.40 ± 0.58	0.18 ± 0.20	0.39 ± 0.33*
GT						
Cervical	0.64 ± 0.20	0.57 ± 0.26	0.53 ± 0.29	0.48 ± 0.25	0.40 ± 0.17	0.39 ± 0.21
Middle	0.60 ± 0.20	0.61 ± 0.20	0.61 ± 0.23	0.61 ± 0.32	0.36 ± 0.23	0.42 ± 0.27

* p ≤ 0.05 (*t*-test). BT = bone thickness, GT = gingival thickness, CEJ-BC = distance from cementoenamel junction to bone crest; GM-CEJ = distance from gingival margin to cementoenamel junction; GM-BC = distance from gingival margin to bone crest.

With regard to age, the GM-CEJ for all tooth groups was significantly smaller in individuals aged ≥50 years than in those aged <50 years (*p*≤ 0.02). The GT for the central incisors was significantly smaller in the older group than in the younger group (*p*= 0.05; Table 3). Other differences were not statistically significant (*p*≥ 0.06).

**Table 3:** Gingival and bone dimensions (in mm) according to age (mean ± standard deviation).

Dimensions	Central incisor		Lateral incisor		Canine	
	< 50 years	≥ 50 years	< 50 years	≥ 50 years	< 50 years	≥ 50 years
GM-BC	3.10 ± 0.92	2.59 ± 0.50	3.54 ±1.29	2.81 ± 0.73	3.15 ± 0.77	2.58 ± 0.68
CEJ-BC	1.89 ± 0.71	2.20 ± 0.50	2.05 ±1.49	2.20 ± 0.86	2.24 ± 1.08	2.96 ± 0.78
GM-CEJ	1.17 ± 0.80	0.33 ± 0.52*	1.49 ± 1.23	0.45 ± 0.76*	0.91 ± 0.99	-0.46 ± 1.06*
BT						
Cervical	0.50 ± 0.22	0.45 ± 0.10	0.47 ± 0.25	0.33 ± 0.26	0.34 ± 0.21	0.38 ± 0.12
Middle	0.56 ± 0.35	0.43 ± 0.28	0.55 ± 0.42	0.53 ± 0.55	0.43 ± 0.36	0.49 ± 0.33
Apical	0.33 ± 0.32	0.23 ± 0.28	0.26 ± 0.51	0.58 ± 0.72	0.24 ± 0.24	0.38 ± 0.44
GT						
Cervical	0.65 ± 0.22	0.47 ± 0.16*	0.53 ± 0.27	0.39 ± 0.23	0.39 ± 0.19	0.38 ± 0.16
Middle	0.63 ± 0.18	0.50 ± 0.23	0.63 ± 0.27	0.46 ± 0.17	0.38 ± 0.26	0.40 ± 0.19

* p ≤ 0.05 (*t*-test). BT = bone thickness, GT = gingival thickness, CEJ-BC = distance from cementoenamel junction to bone crest; GM-CEJ = distance from gingival margin to cementoenamel junction; GM-BC = distance from gingival margin to bone crest.

### RELATIONSHIPS BETWEEN TOOTH INCLINATION AND PERIODONTAL DIMENSIONS

In the central incisor group, tooth inclination was positively and significantly related to the apical BT (R = 0.34, *p*= 0.001). In the canine group, tooth inclination was negatively and significantly related to the cervical BT (R = −0.34, *p*= 0.01) and positively related to the apical BT (R = 0.36, p = 0.01) and negatively related to the GM-CEJ distance (R = -0.31, *p*= 0.03). The other correlations were not statistically significant (*p*≥ 0.06, Table 4).

**Table 4:** Correlation (R) between tooth inclination and gingival and bone dimensions.

Dimensions	Correlation coefficient (R)		
	Central incisors n = 52	Lateral incisors n = 55	Canines n = 53
GM-BC	0.13	-0.24	-0.11
CEJ-BC	0.25	-0.05	0.27
GM-CEJ	0.12	-0.19	-0.31**
Cervical GT	-0.04	0.05	0.20
Cervical BT	0.13	0.04	-0.34*
Middle GT	0.29	0.17	0.17
Middle BT	0.12	0.09	0.14
Apical BT	0.34*	0.12	0.36**

* p ≤ 0.05 (Pearson correlation test); ** p ≤ 0.05 (partial correlation test). GM-BC = distance from gingival margin to bone crest, CEJ-BC = distance from cementoenamel junction to bone crest; GM-CEJ = distance from gingival margin to cementoenamel junction, Cervical GT = gingival thickness at the cervical root third, Cervical BT = bone thickness at the cervical root third, Middle GT = gingival thickness at the middle root third, Middle BT = bone thickness at the middle root third, Apical BT = bone thickness at the apical root third.

## DISCUSSION

The present study found a direct association between central incisor and canine inclinations and BT in the apical third of the root. Furthermore, the inclination of the canines was negatively and significantly related to the cervical BT and positively related to the GM-CEJ. Nevertheless, in the lateral incisors, tooth inclination was not associated with bone or gingival dimensions. Therefore, the tested hypothesis was partially accepted. 

The present study demonstrated that greater labial inclination (proclination) was associated with greater BT in the apical region of the central incisors and canines, in agreement with previous reports on central incisors.^14^ ^,^ ^26^ Conversely, in the present investigation, greater labial inclination of canines was associated with smaller BT in the cervical region. Previous studies also found reduced BT for the maxillary canines, and speculated that this finding was associated with greater prominence of their roots.^7^ ^,^ ^26^ ^-^ ^28^ However, those studies did not evaluate the impact of tooth inclination. In divergence to the findings of the present study, the results of two previous studies did not reveal significant differences in the cervical BT according to the tooth inclination for maxillary central incisors.^13^ ^,^ ^14^ In contrast, another study described that retroinclined maxillary central incisors showed thinner bone at the cervical level than did normal or proclined central incisors.^16^ The increased BT in the apical region and decreased BT in the cervical region of more proclined central incisors and canines can be explained by the retroposition of the tooth apices and anteroposition of the cervical area, due to the tooth inclination. 

In the current study, there was a significant direct association between tooth inclination and the GM-CEJ distance for the canines. In a systematic review, most studies showed that the incidence or severity of gingival recession was greater for more proclined teeth than for less proclined or orthodontically untreated teeth.^11^ However, it should be noted that there are no high-quality studies regarding this topic, and the low level of evidence in the studies included in the former review and the present results indicate that further clinical studies controlling for dental plaque amount are necessary to clarify the relationship between tooth inclination and the GM position.

In the present study no association between GT and maxillary anterior tooth inclination was found, in accordance with the results of previous studies.^10^ ^,^ ^12^ ^,^ ^15^ Although these investigations assessed the buccolingual inclination of the maxillary central incisors through cephalometric analysis and GT through clinical inspection, no relation was found between the two variables. Thus, GT in the maxillary arch seems to be related to factors other than tooth inclination, such as age -as confirmed here-, dental arch location, tooth type, gingival width and inherited condition.^8^ ^,^ ^29^ ^,^ ^30^

The associations between tooth inclination and periodontal dimensions were separately analyzed for each tooth group (central incisors, lateral incisors, and canines) and each third of the root (cervical, middle, and apical thirds), because differences were found in the dimensions according to the tooth group and root segment. The mean gingival and bone thickness and the GM-CEJ distance were significantly smaller in canines compared to the incisors, as observed in previous studies.^7^ ^,^ ^21^ ^,^ ^26^ On the contrary, other studies did not find significant differences in the gingival and bone thickness among the maxillary anterior teeth.^4^ ^,^ ^31^ The discrepancy between studies might be associated with differences in the evaluation technique. While the present study recorded gingival measurements using CBCT images, the former studies used transgingival probing.^4^ ^,^ ^31^

To test for confounders, differences between sexes and age groups were evaluated. Differences in BT and the GM-CEJ distance for the central incisors and canines between men and women were found, as opposed to the findings in previous studies.^26^ ^,^ ^32^ ^,^ ^33^ For the central incisors, women showed thicker bone in the cervical region than men. On the other hand, men showed thicker bone in the middle and apical thirds of canine roots than women. The GM-CEJ was also greater in women than in men. These contrasting results may have occurred because of differences in the methodology and populations studied. With regard to age, the GM-CEJ distance for all tooth groups was significantly smaller in individuals aged ≥50 years than in those aged <50 years, while GT for the central incisors was smaller in the older group. This result is in accordance with a previous investigation and can be attributed to thinning of the epithelium in relation to age.^8^

The strengths of the present study are related to its methodology. CBCT allows simultaneous evaluation of the soft and hard tissues through the use of a single tool; moreover, it shows high accuracy and is a non-invasive and reproducible method.^23^ ^,^ ^24^ The images exhibited a high resolution because they were acquired using a small voxel size. Furthermore, tooth inclination was measured for all maxillary anterior teeth, and the entire labial root surface was screened on axial slices for determining the smallest gingival and bone thickness in the cervical, middle, and apical thirds. Despite the diagnostic advantages, however, it is known that the radiation dose delivered by CBCT is higher than the dose delivered by diagnostic modern digital panoramic and cephalometric imaging combined.^34^

This study also had some limitations. First, the cross-sectional design did not allow obtaining information on the sequence of events, which precludes a conclusion regarding the causal relationship between variables. Second, the sample size was small, had a wide range of chronological age, and no information about the orthodontic or race classifications. Although statistical analysis showed a power of 82% for the association between tooth inclination and gingival thickness, it showed a power of less than 62% for other associations. Furthermore, the sagittal images of 29 teeth were not clear, making 42 measurements impossible. Third, although it has been shown that gingival and bone thickness are not related with the craniofacial morphology,^28^ ^,^ ^35^ the lack of the orthodontic classification for patients in the present sample may be considered a limitation. 

Considering the existence of an association between tooth inclination and bone thickness, evaluation of the periodontium using tomographic images is especially useful for treatment planning, and should be performed particularly when a change in tooth inclination of central incisors and canines is being considered for orthodontic therapy, or the region to be treated already shows thin alveolar bone or evidence of periodontal support loss.^36^

Patients who require orthodontic treatment involving labial movement of the central incisors and canines should undergo a periodontal evaluation before any orthodontic procedure, because the facial alveolar bone of maxillary teeth is thin.^37^ Respecting the biological limits of the patient’s alveolar bone anatomy is of utmost importance to avoid future periodontal problems such as dehiscence.^36^ During orthodontic treatment planning, the final canine position should be given special consideration, because it can affect the soft tissue contour and influence the aesthetic outcome. 

Further research on the effects of orthodontic changes in tooth inclination on the periodontium may provide more information on the cause/effect relationship between tooth inclination and gingival and bone dimensions. 

## CONCLUSIONS

In summary, these results suggest that:

» Greater labial inclination is associated with greater apical BT in the central incisors and canines.
» Greater labial inclination is associated with thinner cervical bone and a smaller GM-CEJ distance in canines.
» For the lateral incisors, there is no correlation between tooth inclination and gingival and bone dimensions.
